# Dynamics of Circulating Follicular Helper and Regulatory T‐Cell Memory Induced by mRNA, Inactivated, and Live‐Attenuated Vaccines

**DOI:** 10.1155/jimr/2293380

**Published:** 2026-07-08

**Authors:** A. China, A. Rahman, L. Hani, M. Kumamoto, Mathieu Surenaud, C. Tcherakian, V. Godot, T. Nogimori, J. D. Lelièvre, Y. Levy, F. Tangy, T. Yamamoto, N. Seddiki

**Affiliations:** ^1^ U955 -Team 16, Faculty of Medicine, INSERM, Créteil, 94000, France, inserm.fr; ^2^ Paris-Est Créteil University, Créteil, 94000, France; ^3^ Vaccine Research Institute (VRI), Créteil, 94000, France, vaccine-research-institute.fr; ^4^ Laboratory of Precision Immunology, Center for Intractable Disease and ImmunoGenomics, National Institutes of Biomedical Innovation Health and Nutrition, Ibaraki, Osaka, 567–0085, Japan; ^5^ Laboratory of Aging and Immune Regulation, Graduate School of Pharmaceutical Sciences, The University of Osaka, Suita, Osaka, 565–0871, Japan, osaka-u.ac.jp; ^6^ Pneumology Department, Foch Hospital, Suresnes, France; ^7^ Simone Veil Research Unit, Versailles Saint-Quentin-en-Yvelines University, Montigny-Le-Bretonneux, France, uvsq.fr; ^8^ Henri Mondor Hospital- A. Chenevier, Service Clinical Immunology and Infectious Diseases Department, Créteil, 94000, France; ^9^ Institut Pasteur - Oncovita Joint Laboratory, Université de Paris Cité, Paris, France; ^10^ Department of Virology and Immunology, Graduate School of Medicine, The University of Osaka, Suita, Osaka, 565–0871, Japan, osaka-u.ac.jp; ^11^ Immune Diseases, Microbiology and Innovative Therapies (IDMIT/UMRS1184), Inserm, Paris-Saclay University, CEA, Fontenay-aux-Roses, Le Kremlin-Bicêtre, France, universite-paris-saclay.fr; ^12^ Institut Hospitalo-Universitaire Comprehensive SEPSIS Center, Paris-Saclay University, Saclay, France, universite-paris-saclay.fr; ^13^ Immunology and Pathogenesis Program, Kirby Institute, UNSW, Sydney, New South Wales, Australia, unsw.edu.au

## Abstract

OX40 signaling promotes the differentiation of memory CD4^+^ T cells into T follicular helper (Tfh) cells that support germinal center (GC) B‐cell responses. Using an extended activation‐induced marker (AIM) assay based on OX40 (CD134) and CD25 coexpression, we quantified antigen‐specific circulating memory Tfh and T follicular regulatory (Tfr) cells following in vitro stimulation with influenza, SARS‐CoV‐2, or measles antigens. Responses were assessed longitudinally in recipients of inactivated influenza (*n* = 20) and mRNA COVID‐19 (*n* = 13) vaccines and cross‐sectionally in individuals vaccinated with the live‐attenuated measles vaccine (*n* = 8). All three vaccines elicited humoral responses. Functional assays, performed when sufficient cell numbers were available, showed that sorted influenza‐specific Tfh cells (OX40^+^CD25^+^CXCR5^+^), cocultured with autologous CD27^+^CD19^+^ memory B cells, promoted B‐cell expansion and antibody secretion. This was accompanied by increased CXCL13, IL‐21, soluble CD40L, and APRIL, consistent with effective T‐cell help and B‐cell activation. Comparative analyses revealed distinct patterns of follicular immune responses: influenza vaccination induced coordinated Tfh and Tfr responses, whereas mRNA COVID‐19 vaccination generated increased Tfh but minimal Tfr cells. At peak response, measles and influenza vaccinees exhibited comparable Tfh and Tfr frequencies, while COVID‐19 vaccinees showed similar Tfh but reduced Tfr proportions. Overall, the extended AIM assay represents a practical and minimally invasive approach for simultaneous monitoring of Tfh and Tfr populations in humans, providing a scalable framework to investigate follicular immune balance across vaccination settings. Beyond enabling sensitive detection of antigen‐specific memory responses, this approach highlights the importance of assessing both helper and regulatory follicular compartments to better understand vaccine‐induced immunity.

## 1. Introduction

Vaccination remains one of the most effective strategies for preventing infectious diseases by inducing robust and durable humoral and cellular immune responses. These immune components act synergistically to neutralize pathogens, eliminate infected cells, and establish an immunological memory that protects against future exposures.

The rapid development and global rollout of mRNA‐based COVID‐19 vaccines highlighted the ability of this platform to generate robust neutralizing antibody responses and strong protection against severe disease [1]. However, several studies have documented a relatively rapid decline in antibody titers following the primary series, with reductions of up to ~ 70% within 6 months of the second dose [1, 2].

Comparative assessments of four SARS‐CoV‐2 vaccine platforms, two mRNA vaccines (mRNA‐1273 and BNT162b2), a viral vector vaccine (Janssen Ad26.COV2.S), and an adjuvanted protein‐subunit vaccine (Novavax NVX‐CoV2373) have examined the kinetics of immune memory in a side‐by‐side manner. These analyses consistently show that although mRNA vaccine recipients experience substantial declines in circulating antibody levels, memory B‐cell and T‐cell responses remain comparatively stable over time [3, 4].

Protection against influenza is not solely reliant on the humoral (antibody‐mediated) response. High levels of HA‐specific antibodies are associated with reduced risk and severity of infection, but they do not provide complete sterilizing immunity, and breakthrough infections can still occur [5]. The immune response against influenza viruses also relies on other aspects of the immune system, particularly cellular immunity, which includes B cells and T cells [6–10]. In the absence of antibody‐mediated immunity, preexisting memory B cells [11, 12] and broadly cross‐reactive memory T cells [13–15] can be recalled and protect against influenza.

Live‐attenuated vaccines, such as the measles vaccine, represent yet another paradigm of vaccine‐induced immunity. By mimicking natural infection and sustaining antigen presentation, they promote strong germinal center (GC) activity, driving the generation of long‐lived plasma cells and memory B cells. Consequently, antibody responses induced by live‐attenuated vaccines can persist for decades [16, 17].

The quality and longevity of humoral immunity are intimately linked to the magnitude and regulation of GC responses within secondary lymphoid tissues. These responses are orchestrated by two specialized subsets of CD4^+^ T cells: T follicular helper (Tfh) cells, which promote B‐cell activation, affinity maturation, and differentiation into memory B cells or antibody‐secreting plasma cells, and T follicular regulatory (Tfr) cells, which express a range of proteins that are typical of regulatory T (Treg) cells and restrain excessive or autoreactive GC reactions to maintain immune tolerance and ensure appropriate immune responses [18, 19]. Preclinical studies in mice have shown that Tregs are involved in facilitating Tfh differentiation, guiding immune cell migration to infection sites, and mitigating tissue inflammation during the resolution phase of infection [20–22]. Thus, maintaining the balance between immune cell populations is crucial for effective vaccine responses.

Direct assessment of Tfh and Tfr cells in humans is limited by their localization in the GCs of secondary lymphoid organs, such as lymph nodes and the spleen, which are not easily accessible in routine clinical studies. Recent technological advances such as single‐cell transcriptomics and fine‐needle aspiration (FNA) of lymph nodes have provided valuable insights into the cellular dynamics of vaccination at the site of immune priming [23, 24]. Nevertheless, these approaches are resource‐intensive, invasive, and not suitable for large‐scale immuno‐monitoring in clinical trials or population studies. Consequently, we rely on their circulating counterparts, Tfh and Tfr cells, found in the peripheral blood. These cells share key phenotypic markers and some functional attributes with their tissue‐resident equivalents [25, 26]. While most human studies have focused on Tfh responses, the regulatory component remains underexplored despite its essential role in controlling GC activity and maintaining antibody quality [18, 19]. In this study, we employed an extended activation‐induced marker (AIM) assay to detect and characterize phenotypically and functionally memory antigen‐specific Tfh and Tfr cells in peripheral blood. We then applied this method to compare the frequency and dynamics of these CD4^+^ T cell subsets across three vaccine types administered to healthy individuals: inactivated influenza, mRNA COVID‐19, and live‐attenuated measles.

## 2. Materials and Methods

### 2.1. Study Design

All subjects were recruited in their respective groups after checking their vaccination records and having their written consent.

#### 2.1.1. Influenza

Twenty healthy volunteers aged 30–40 years were immunized with the seasonal inactivated influenza vaccine (VAXIGRIP or INFLUVAC). Blood draws were collected at baseline (day 0, prevaccination) and on days 3, 7, 14, and 28 following immunizations. All participants provided written informed consent, and study protocols were approved by the institutional Hamburg and Foch hospital ethics committees, respectively.

#### 2.1.2. COVID‐19

A total of 13 participants were comprised of healthy Japanese adults with an age range of 44–70 years who had previously received two doses of the mRNA vaccine BNT162b2 (Pfizer‐BioNTech) at an interval of 6–12 months prior to the study’s commencement. Blood samples were collected at the following time points: prior to the third BNT162b2 vaccination (baseline or preboost), 2 weeks after the third dose (2 weeks postboost), 3 months after the third dose (3 months postboost), and 6 months after the third dose (6 months postboost). All participants provided written informed consent, and the study protocol was approved by the Japanese institutional ethics committee (Approval Number 279‐02).

#### 2.1.3. Measles

The cohort comprises subjects born after 1980’s (age range 19–32 years) who were vaccinated during infancy (1–2 years old) and vaccinated with measles vaccine (*n* = 8). Cryopreserved peripheral blood mononuclear cells (PBMCs) were obtained from the Institut Pasteur (study approved by the institutional ethics committee, Institut Pasteur Paris).

### 2.2. PBMCs and Serum Collections

PBMCs and serum samples were collected from fresh blood of (i) 20 healthy individuals at baseline and on days 3, 7, 14, and 28 following immunization with the seasonal inactivated influenza vaccine (Foch Hospital, Suresnes, France, and the University Medical Center Hamburg‐Eppendorf, Germany); (ii) 13 COVID‐19 vaccinated individuals (National Institutes of Biomedical Innovation, Health and Nutrition, Japan) prior to the third BNT162b2 vaccination (preboost), 2 weeks postboost, 3 months postboost, and 6 months postboost; and (iii) eight individuals born after 1980 and who received a measles vaccine during infancy (12–18 months). All samples were cryopreserved in liquid nitrogen until use.

### 2.3. Ex Vivo Phenotyping

Thawed PBMCs were centrifuged twice at 1400 rpm for 8 min, cell pellets were resuspended in 30 µL of antibody cocktails targeting either B cell, and incubated for 30 min at 4°C in the dark. The B‐cell panel included VIVID AmCyan viability dye (Thermo Fisher), anti‐CD3 AF700, anti‐CD19 PeCF594, anti‐CD38 PerCP‐Cy5.5 (BioLegend), anti‐CD27 APC, anti‐CD21 BV711, and anti‐CD39 PeCy7 (eBioscience).

### 2.4. Cell Culture and OX40‐AIM Assay

For influenza and measles, thawed PBMCs were cultured in Iscove’s modified Dulbecco’s medium (IMDM) supplemented with 10% heat‐inactivated human AB serum and 10,000 U/100 mL penicillin‐streptomycin. Cells were plated at 2 × 10^6^ cells/mL in 24‐well plates and stimulated with 1.5 μg/mL of VAXIGRIP 2016/2017 influenza vaccine or measles virus (MeV) antigen, Edmonston strain (#PR‐BA102‐S‐L, Jena Bioscience) at 1 µg/mL, respectively. Controls included unstimulated cells and cells stimulated with 1 μg/mL staphylococcal enterotoxin B (SEB). Cultures were incubated at 37°C with 5% CO_2_ for 44 h. At 39 h, GolgiPlug and GolgiStop (BD Biosciences) were added to inhibit cytokine secretion. After incubation, cells were stained using the “Stimulation” panel, including VIVID AmCyan, anti‐CD3 AF700, anti‐CD4 BV605, anti‐CD25 BV421, and anti‐CD39 BV711 (all from BioLegend), anti‐CD8 APC‐H7 (Miltenyi), anti‐CXCR5 AF488, and anti‐CD134 PE (from BD Biosciences), and intracellular markers anti‐T‐bet PerCP‐Cy5.5, anti‐FoxP3 PeCF594, anti‐Bcl6 PeCy7, and anti‐IL‐21 AF647 (from BD Bioscences). The cells were then analyzed using a BD FACSARIA flow cytometer (BD Biosciences) and FlowJo v.10.8.1. Frequencies of influenza‐ or MeV‐specific Tfh and Tfr cells were calculated by subtracting the background of unstimulated samples. Positive responses were defined if there was a reactivity of 0.01% or more after background subtraction.

To analyze SARS‐CoV‐2 spike‐specific T cells, PBMCs were incubated in 1 mL of RPMI 1640 medium containing 50 U/mL benzonase nuclease (Millipore, Darmstadt, Germany), 10% fetal bovine serum, and penicillin‐streptomycin for 1 h. Next, cells were incubated in 200 µL of medium with or without peptides (17‐mers overlapping by 10 residues) corresponding to the full‐length SARS‐CoV‐2 spike, at a final concentration of 2 µg/mL of each peptide, for 44 h. Thereafter, 0.2 µL BD GolgiPlug and 0.14 µL BD GolgiStop (both from BD Biosciences) were added and incubated for 6 h. After incubation, cells were stained using Fixable Viability Stain 5775V (BD Biosciences), anti‐CD45RA‐BB515 (L48, BD Biosciences), anti‐CD134‐BB700 (L106, BD Biosciences), anti‐CD3‐APC‐H7 (SP34−2, BD Biosciences), anti‐CD4‐BUV496 (L200, BD Biosciences), anti‐CD8‐BUV563 (RPA‐T8, BD Biosciences), anti‐CD27‐BUV661 (O323, BD Biosciences), anti‐CD45RO‐BUV805 (UCHL1, BD Biosciences), anti‐CD127‐PE‐Cy5 (R34.34, Beckman Coulter Life Sciences), anti‐CXCR5‐PE‐CF594 (MUSUBEE, eBioscience), anti‐CD25‐AF700 (BC96, BioLegend), anti‐CCR7‐BV650 (G043H7, BioLegend), and anti‐PD‐1‐BV786 (EH12‐2H7, BioLegend) antibodies. After fixation and permeabilization using a Transcription Factor Buffer Set (BD Biosciences), the cells were stained with anti‐Bcl6‐PE (K112−91, BD Biosciences), anti‐FoxP3‐APC (236A/E7, BioLegend), and anti‐IFNγ‐BV421 (4 S.B3, BioLegend) antibodies. The cells were then analyzed using a BD FACSymphony A5SE flow cytometer (BD Biosciences) and FlowJo v.10.8.1. Frequencies of spike‐specific Tfh and cTfr cells were calculated by subtracting the background of unstimulated samples (DMSO). Positive responses were defined if there was a reactivity of 0.01% or more after background subtraction.

### 2.5. Cell Sorting and Coculture Assays

To increase the yield of antigen‐specific Tfh cells, PBMCs from multiple time points of the same donor (*n* = 3) were pooled. Cells were seeded at 12 × 10^6^ cells/5 mL per well in 6‐well plates and stimulated using the OX40 assay without control wells. After 44 h, cells were stained with the “Sorting” panel: anti‐CD3 AF700, anti‐CD27 PE, anti‐CD134 BV421, anti‐CXCR5 AF488, anti‐CD19 PeCF594 (BD), anti‐CD4 APC‐Vio770, and anti‐CD25 APC (Miltenyi). The sorted populations included memory B cells (CD3^−^CD4^−^CD19^+^CD27^+^), nonspecific CD4^+^ T cells (CD25^+^CD134^−^), antigen‐specific CD4^+^ T cells (CD25^+^CD134^+^CXCR5^−^), and follicular helper‐like CD4^+^ T cells (CD25^+^CD134^+^CXCR5^+^). Cells were rested for 24 h in RPMI supplemented with 10% human AB serum, 1% penicillin/streptomycin, and 100 IU/mL IL‐2, counted, and seeded into 96‐well plates at 20,000 cells per 200 µL per well in a 1:1 ratio with memory B cells. Cocultures were maintained in RPMI with penicillin/streptomycin, puromycin, 10% FBS, and 100 ng/mL SEB [27]. At harvest, 50 µL of supernatant was collected for Luminex cytokine profiling and 100 µL was kept for hemagglutinin‐specific antibody measurement.

### 2.6. Hemagglutination Inhibition (HAI) Assay Using Coculture Supernatants

HAI assays were performed on coculture supernatants following the standardized protocol established by Dr V. Enouf (Institut Pasteur), adapted for cell culture fluids. Briefly, supernatants were serially diluted two‐fold in V‐bottom microplates and incubated with four hemagglutination units of the influenza virus for 30 min at room temperature. Subsequently, 0.5% turkey red blood cells were added, and plates were incubated for 30 min at 4°C. The highest dilution inhibiting hemagglutination was recorded as the HAI titer, reflecting functional influenza‐specific antibody activity in the coculture supernatants. This adaptation maintains assay sensitivity and specificity consistent with established serological testing methods.

### 2.7. ELISA for MeV Titer Measurement

MeV antigen at 1 µg/mL in carbonate buffer, was coated overnight at 4°C onto 96‐well plates (#439454, Thermo Scientific) and then blocked for 1 h at 37°C with a saturation buffer (PBS, 0.05% Tween, 3% BSA). Sera samples were serially diluted (PBS, 0.05% Tween, 1% BSA) and incubated on plates for 1 h at 37°C. After washing steps (0.05% Tween in PBS), a secondary (H&L) peroxidase‐conjugated goat anti‐human IgG antibody (#115‐035‐146, Jackson Immuno Research) 2 mg/mL was added at a dilution of 1/5000 for 1 h at 37°C. Antibody binding was revealed by addition of the TMB substrate (#5120‐0047, Euro bio), and the reaction was stopped by the addition of H_2_SO_4_ 1 M. The optical densities (ODs) were recorded at 450 nm. The endpoint titers for each individual serum were calculated as the reciprocal of the last dilution, giving twice the absorbance of negative control sera.

### 2.8. ELISA for Anti‐SARS‐CoV‐2 Receptor‐Binding Domain (RBD) IgG Titer Measurement

Plasma concentrations of total IgG antibodies specific for the SARS‐CoV‐2 spike RBD were quantified by ELISA. Recombinant spike RBD proteins representing Wuhan‐1, BA.1, BA.2, BA.5, BQ.1.1, XBB, BA.4.6, and BA.2.75 variants were purchased from SinoBiological (Beijing, China). For the assay, 96‐well plates were coated with RBD protein and incubated overnight at 4°C. Plates were then washed, blocked for 1 h with blocking buffer, washed again, and incubated with diluted plasma samples for 2 h at 25°C. After washing, plates were incubated for 1 h with biotinylated anti‐human total IgG (BD Biosciences, San Jose, CA, USA), followed by another wash and a 1 h incubation with horseradish peroxidase‐conjugated streptavidin (Thermo Fisher Scientific, Waltham, MA, USA) at 25°C. Color development was achieved by adding TMB peroxidase substrate (KPL, Gaithersburg, MD, USA), and the reaction was stopped after 10 min with 2 mol/L H_2_SO_4_. OD at 450 nm was measured using an Epoch 2 Microplate Spectrophotometer (Agilent, Santa Clara, CA, USA). Endpoint titers were calculated using a cut‐off OD value of 0.3.

### 2.9. Luminex Multiplex Cytokine Assay

Cytokine quantification of 18 analytes (APRIL, CXCL13, IFN‐γ, IL‐10, IL‐12p70, IL‐13, IL‐17 A, IL‐17 F, IL‐2, IL‐21, IL‐22, IL‐23, IL‐27, IL‐4, IL‐6, IP‐10, sCD40L, and TNF‐α) was performed using the ProcartaPlex Mix & Match Human 18‐plex kit (Invitrogen, Thermo Fisher), following manufacturer instructions. A 9‐point standard curve was generated by serial 1:4 dilutions. Magnetic beads (50 µL per well) were incubated with standards, samples, or blanks for 2 h at room temperature with agitation. After washing, 25 µL of detection antibody mix was added and incubated for 30 min, followed by the addition of 50 µL streptavidin‐PE and a further 30 min incubation. After the final washes, beads were resuspended in reading buffer, shaken for 5 min, and acquired on a Bio‐Plex 200 system.

### 2.10. Statistical Analysis

Graphical representations and statistical analyses were performed using GraphPad Prism 9.0 software (Graph Pad Software Inc.). Results were expressed as the median percentage frequency. Group differences were assessed using the Friedman test followed by Dunn’s multiple‐comparison test, the Kruskal–Wallis test followed by Dunn’s multiple‐comparison test, or the Mann–Whitney test where appropriate.  ^∗^ *p*  < 0.05,  ^∗∗^ *p*  < 0.01,  ^∗∗∗^ *p*  < 0.001 indicate statistical significance.

## 3. Results

### 3.1. Influenza, COVID‐19, and Measles Vaccinations Induce Humoral Responses

**Figure 1:**
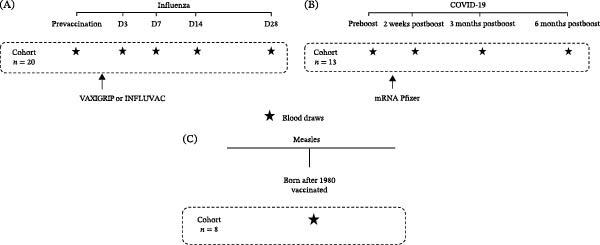
Schematic overview of the influenza, COVID‐19, and measles vaccine cohorts. (A) 20 healthy volunteers aged 30–40 years (already exposed and/or vaccinated against flu) were immunized with the seasonal inactivated influenza vaccine (VAXIGRIP or INFLUVAC). Blood draws (stars) were collected at baseline (day 0, prevaccination) and on days 3, 7, 14, and 28 following immunization. (B) COVID‐19: a total of 13 participants were comprised of healthy Japanese adults with an age range of 44–70 years who had previously received two doses of the mRNA vaccine BNT162b2 (Pfizer‐BioNTech) at an interval of 6–12 months prior to the study’s commencement. Blood samples were collected at the following time points: prior to the third BNT162b2 vaccination (baseline or preboost), 2 weeks after the third dose (2 weeks postboost), 3 months after the third dose (3 months postboost), and 6 months after the third dose (6 months postboost). (C) Measles: the cohort comprises subjects born after 1980’s (age range 19–32 years) and vaccinated with measles vaccine during infancy (*n* = 8). Cryopreserved PBMCs were obtained from these individuals.

We included three cohorts of healthy individuals vaccinated with either the inactivated influenza vaccine, an mRNA COVID‐19 vaccine, or the live‐attenuated MeV vaccine (Figure 1).

To confirm serological and vaccination status, we measured antibody responses in all cohorts. As expected, individuals vaccinated with MeV during infancy exhibited antibody titers comparable to those observed in measles‐infected control individuals (26,559.89 ± 11,582.19 vs. 30,474 ± 10,432.67 mIU/mL; data not shown), confirming effective immunization and long‐term memory.

**Figure 2:**
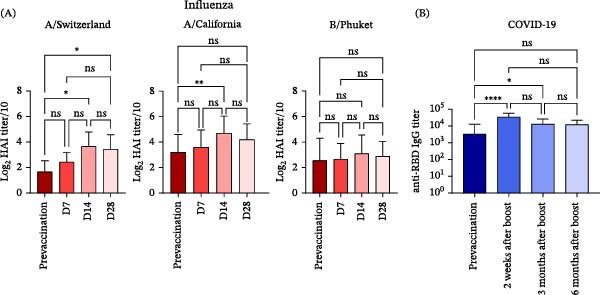
(A) Antibody responses in individuals vaccinated with either influenza (VAXIGRIP or INFLUVAC) vaccine. (B) Anti‐RBD IgG endpoint titers over time in plasma samples obtained from BNT162b2‐vaccinated group differences were assessed using the Friedman test followed by Dunn’s multiple‐comparison test. Statistical significance was determined based on *p*‐values: *p* < 0.05 ( ^∗^), *p* < 0.01 ( ^∗∗^), and *p* < 0.001 ( ^∗∗∗^); ns indicates no statistically significant difference (*p* ≥ 0.05).

Influenza‐specific antibody responses in serum samples from a cohort of 20 healthy adult volunteers immunized with inactivated seasonal influenza vaccines (VAXIGRIP or INFLUVAC) were observed. All individuals showed increased IgG titers by day 28 postvaccination compared with baseline, indicating seroconversion consistent with vaccine uptake (Figure 2A). In the influenza cohort, IgG responses at day 14 were significantly increased for A/Switzerland and A/California, whereas no significant change was observed for B/Phuket. At day 28, a significant increase compared with the baseline was maintained only for A/Switzerland. In the COVID‐19 cohort, IgG titers increased following the second and third booster doses relative to prevaccination levels, consistent with progressive boosting of the humoral response.

In parallel, we quantified circulating memory B cells (CD19^+^CD27^+^CD38^+^) to assess early B‐cell dynamics postimmunization. The frequency of this population peaked at day 7 following vaccination (Figure S1), in line with previously reported kinetics of vaccine‐induced memory B‐cell responses [26, 28]. These data confirm that inactivated influenza vaccination elicits a rapid humoral response, characterized by early memory B‐cell expansion followed by stable antibody titers.

To assess immune responses to the COVID‐19 mRNA vaccine, we enrolled 13 healthy adults who had received the Pfizer BNT162b2 mRNA vaccine and collected samples at four timepoints: prior to the third BNT162b2 dose (preboost), 2 weeks after the third dose (2 weeks postboost), 3 months after the third dose (3 months postboost), and 6 months after the third dose (6 months postboost). First, we measured anti‐spike RBD IgG endpoint titers against SARS‐CoV‐2 WT in plasma using an ELISA. Compared with preboost, anti‐RBD IgG antibody titers increased significantly 2 weeks postboost (median baseline = 621.22, median 2 weeks postboost = 37522.27; *p* = 0.0002, Figure 2B). Although titers at 3 months and 6 months postboost were significantly lower than at 2 weeks, they remained significantly higher than preboost values (median = 8632.85 at 3 months postboost, median = 10658.41 at 6 months postboost; *p* = 0.0046 between preboost and 3 m postboost, *p* = 0.0134 between preboost and 6 months postboost, *p* = 0.0005 between 2 weeks postboost and 3 months postboost, *p* = 0.0024 between 2 weeks postboost and 6 months postboost, Figure 2B).

### 3.2. Phenotypic and Functional Characterization of Circulating Tfh and Tfr Cells

**Figure 3:**
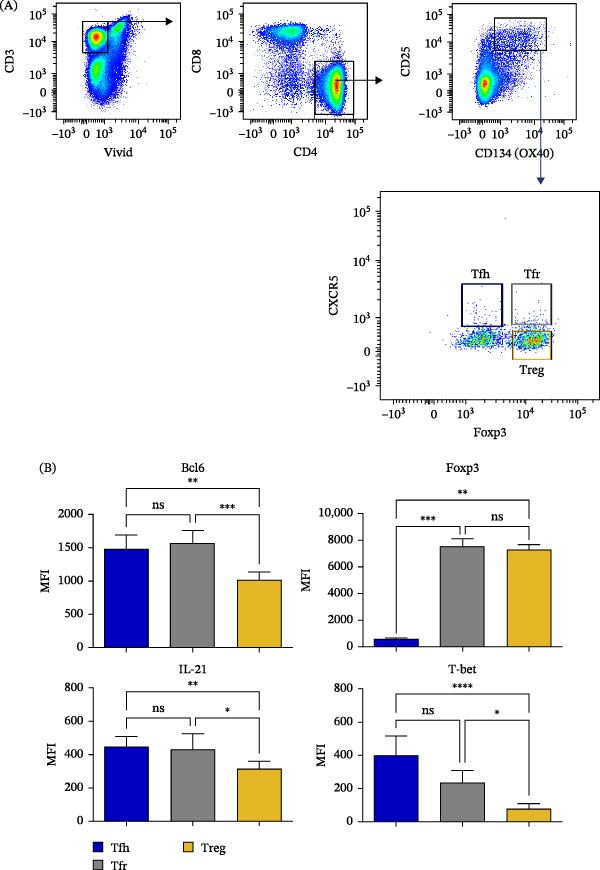
(A) Gating strategy for identification of antigen‐specific CD25^+^CD134^+^ cells and then depict Tfh, Tfr, and Tregs subsets. (B) Bcl6, Foxp3, IL‐21, and T‐bet mean fluorescence intensity (MFI) have been determined using flow cytometry. Statistical significance was determined based on *p*‐values: *p* < 0.05 ( ^∗^), *p* < 0.01 ( ^∗∗^), and *p* < 0.001 ( ^∗∗∗^); ns indicates no statistically significant difference (*p* ≥ 0.05).

Given the early detection of IgG responses, we hypothesized that influenza‐specific memory Tfh cells would also be detectable in peripheral blood by day 7 postvaccination. To identify these cells, we employed the OX40‐AIM assay [29–31], following a 44 h in vitro stimulation of PBMCs with the inactivated influenza vaccine (INFLUVAC). The gating strategy used to delineate Tfh, Tfr, and Treg is shown in Figure 3A.

Flow cytometric analysis revealed a distinct population of activated CD4^+^OX40^+^CD25^+^CXCR5^+^ T cells expressing Bcl6 but not Foxp3, consistent with a circulating memory Tfh phenotype (Figure 3B and Figure S2). In addition, we identified Tfr cells as CXCR5^+^Bcl6^+^Foxp3^+^, and Tregs as CXCR5^+^Bcl6^-^Foxp3^+^. The expression of key transcription factors, Bcl6, Foxp3, and T‐bet, along with intracellular IL‐21 production, confirmed the functional identity of each subset (Figure 3B). Indeed, Tfh cells are functionally heterogeneous and can be further subdivided into Tfh1, Tfh2, and Tfh17 subsets based on their transcriptional profiles and cytokine signatures [25, 26, 32]. In our study, influenza‐specific Tfh cells expressed high levels of T‐bet, indicating a Tfh1 polarization consistent with type 1 antiviral immunity. These findings underscore the utility of the OX40‐AIM assay in capturing functionally distinct antigen‐specific CXCR5^+^CD4^+^ T‐cell subsets postvaccination.

### 3.3. Influenza‐Specific Tfh Cells Promote Antibody Production in B‐Cell Cocultures

**Figure 4:**
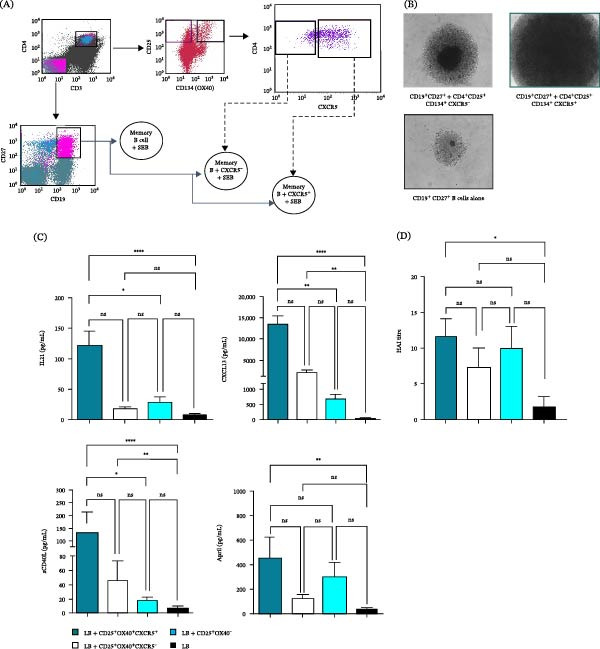
(A) Gating strategy for Tfh and B cell sorting. (B) Microscopic analysis showing cell proliferation and density in cocultures of B cell with the different sorted Tfh CD25^+^CD134^+^CXCR5^+^ and non‐Tfh populations CD25^+^CD134^+^CXCR5^−^ and CD25^+^CD134^−^ (same magnification x50; an example of one representative individual). (C) CXCL13, sCD40L, IL‐21, and APRIL in supernatants from cocultures at day 6 from (B). (D) Antibody activity was evaluated using HAI assay, which quantifies the ability of antibodies to block hemagglutinin‐mediated red blood cell agglutination detection in the supernatant collected from of each coculture condition from (B). Group differences were assessed using the Kruskal–Wallis test.  ^∗^ *p*  < 0.05,  ^∗∗^ *p*  < 0.01,  ^∗∗∗^ *p*  < 0.001 indicate statistical significance; ns indicates no statistically significant difference (*p* ≥ 0.05).

To assess the functional capacity of influenza‐specific Tfh cells, we performed ex vivo coculture assays using PBMCs from influenza‐vaccinated individuals. FACS‐sorted CD4^+^OX40^+^CD25^+^CXCR5^+^ Tfh and CD4^+^OX40^+^CD25^+^CXCR5^−^ non‐Tfh T cells, from three individuals, were cocultured for 6 days with autologous CD3^−^CD19^+^CD27^+^ memory B cells (LB) in the presence of SEB, used as a strong stimulus for these sorted pure cells as previously described [27]. Control conditions included CD4^+^OX40^−^CD25^+^cells in the presence of LB and LB cells cultured alone (Figure 4A).

Microscopic analysis of the cocultures showed that wells containing B cells with CXCR5^+^CD25^+^OX40^+^ influenza‐specific Tfh cells displayed markedly higher cell density than wells containing B cells with CXCR5^-^CD25^+^OX40^+^ or with CD4^+^OX40^+^CD25^+^ non‐Tfh cells. Given that all cultures were initiated with the same number of cells (20,000; 1:1 ratio), this increased density indicates substantially greater cell proliferation in the presence of CXCR5^+^CD25^+^OX40^+^ influenza‐specific Tfh cells (Figure 4B). These findings suggest that CXCR5^+^CD25^+^OX40^+^ memory Tfh cells possess a superior capacity to support B‐cell survival and proliferation.

To assess functional activity, culture supernatants were analyzed for canonical Tfh‐associated factors using a Luminex assay. CXCL13, IL‐21, soluble CD40 ligand (sCD40L), and APRIL (TNFSF13), key mediators of GC activity and humoral immunity [27], were significantly elevated in CXCR5^+^CD25^+^OX40^+^ influenza‐specific Tfh‐B cell cocultures compared with wells containing non‐Tfh (Figure 4C). Notably, APRIL, a cytokine that promotes memory B‐cell and long‐lived plasma‐cell survival [33], was also significantly increased in the CXCR5^+^CD25^+^OX40^+^ influenza‐specific Tfh‐B condition. Cytokines associated with Th1 and Th17 responses were also similarly elevated under these conditions (data not shown).

Influenza‐specific antibody production was assessed using the HAI assay, which measures the capacity of antibodies to inhibit hemagglutinin‐mediated red blood cell agglutination and is routinely used by influenza reference centers to evaluate seroconversion after infection or vaccination. Slightly higher HAI titers were observed in cocultures containing CXCR5^+^CD25^+^OX40^+^ influenza‐specific Tfh cells with autologous B cells compared with non‐Tfh cocultures, although the difference did not reach statistical significance, likely due to the small sample size.

Altogether, these findings demonstrate that influenza‐specific CXCR5^+^CD25^+^OX40^+^ Tfh cells effectively drive B‐cell proliferation and survival and to a lesser extent the production of virus‐specific antibodies.

### 3.4. Distinct Kinetics of Tfh and Tfr Responses Following Inactivated Influenza or mRNA COVID‐19 Vaccination

**Figure 5:**
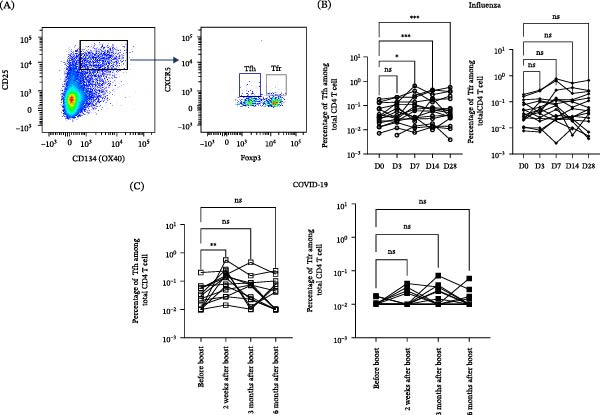
(A) Gating strategy for circulating Tfh and Tfr specific‐memory populations. Antigen‐specific cells are identified by the AIM assay based on antigen‐induced upregulation of CD25 and CD134 (OX40) and Tfh and Trf are defined based on expression of CXCR5 and Foxp3. Longitudinal follow‐up of influenza and spike‐specific Tfh and Tfr frequencies in total CD4^+^ T cells (B and C). Group differences were assessed using the Friedman test followed by Dunn’s multiple‐comparison test. Statistical significance was determined based on *p*‐values: *p* < 0.05 ( ^∗^), *p* < 0.01 ( ^∗∗^), and *p* < 0.001 ( ^∗∗∗^); ns indicates no statistically significant difference (*p* ≥ 0.05).

We next performed a longitudinal analysis of Tfh and Tfr responses in the influenza and COVID‐19 vaccine cohorts (Figure 5A).

In individuals immunized with the inactivated seasonal influenza vaccine, circulating Tfh and Tfr cell populations showed broadly similar kinetics over time (Figure 5B). Both subsets increased and peaked around day 7 postvaccination (*p*  < 0.05 for Tfh; *p*  > 0.05 for Tfr), followed by a decrease at day 14 (*p*  > 0.05 for both subsets). At day 28, Tfh cells showed a modest increase (*p*  < 0.001), whereas Tfr frequencies remained relatively stable (*p*  > 0.05). This pattern is consistent with the expected temporal dynamics of GC responses, where Tfh cells support B‐cell responses and Tfr cells contribute to the regulation of GC activity [19].

**Figure 6:**
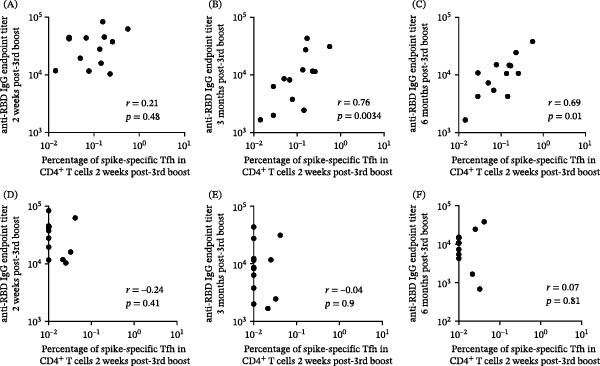
(A) Correlation between spike‐specific Tfh cell frequency and anti‐RBD IgG endpoint titer at 2 weeks after 3rd mRNA vaccination. (B) Correlation between spike‐specific Tfh cell frequency and anti‐RBD IgG endpoint titer at 3 months after 3rd mRNA vaccination. (C) Correlation between spike‐specific Tfh cell frequency and anti‐RBD IgG endpoint titer at 6 months after 3rd mRNA vaccination. (D) Correlation spike‐specific cTfr cell frequency and anti‐RBD IgG endpoint titer at 2 weeks after 3rd mRNA vaccination. (E) Correlation spike‐specific cTfr cell frequency and anti‐RBD IgG endpoint titer at 3 months after 3rd mRNA vaccination. (F) Correlation spike‐specific cTfr cell frequency and anti‐RBD IgG endpoint titer at 6 months after 3rd mRNA vaccination. Correlations were calculated using the nonparametric Spearman’s rank test (*n* = 13). Dashed lines indicate the best‐fit lines calculated by log–log nonlinear regression.

In contrast, analysis of a cohort of individuals (*n* = 13) who received mRNA‐based COVID‐19 vaccines revealed divergent follicular dynamics. Tfh cell frequencies increased following the second vaccine dose (median pre = 0.024, median 2 weeks after boost = 0.135, *p* = 0.0038, Figure 5C), typically peaking 2 weeks after the booster, consistent with helper T cell priming [34, 35]. However, Tfr cell frequencies remained low and largely unchanged, resulting in a skewed Tfh: Tfr ratio (Figure 5C). Since Tfh cells are known to play a critical role in supporting B‐cell differentiation and antibody production, we next investigated whether the magnitude of the spike‐specific Tfh response was associated with subsequent antibody titers. The frequency of spike‐specific Tfh cells at 2 weeks postboost showed a positive trend, but the correlation with anti‐RBD IgG titers at the same time point did not reach statistical significance (*r* = 0.2143, *p* = 0.4819, Figure 6A). Notably, the frequencies of spike‐specific Tfh cells at 2 weeks postboost were significantly correlated with RBD‐specific IgG titers measured at both 3 months (*r* = 0.7637, *p* = 0.0034, Figure 6B) and 6 months (*r* = 0.6933, *p* = 0.0106, Figure 6C) after boost. In contrast, Tfr cells were only modestly induced following the third vaccination, and no clear correlations were observed between Tfr frequencies and anti‐RBD IgG titers at any of the examined time points (not shown).

### 3.5. Comparative Analysis Across Influenza, COVID‐19, and Measles Vaccine Cohorts

**Figure 7:**
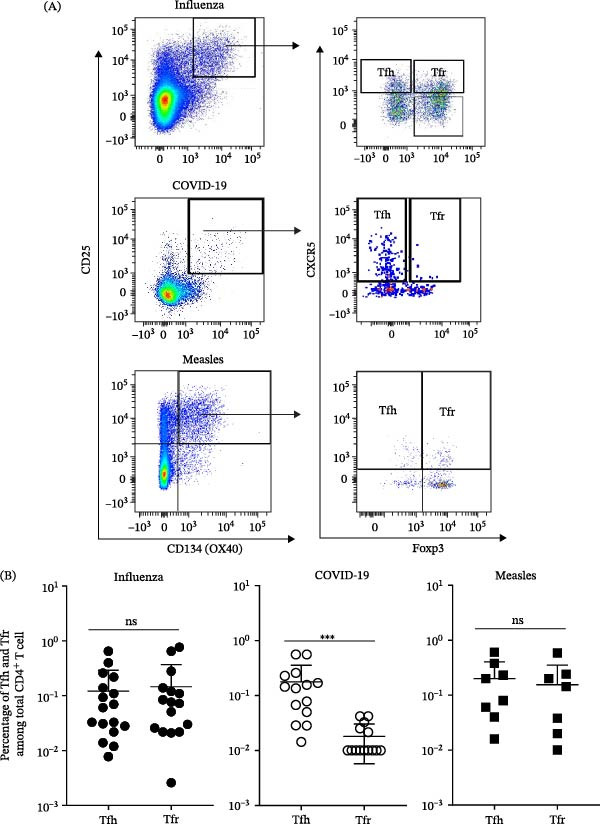
(A) Gating strategy for memory specific Tfh and Tfr in peripheral blood of influenza, COVID‐19, and measles immunized individuals. (B) Tfh and Tfr comparison at peak response for each of the three vaccines. Group comparisons were performed using the Mann–Whitney *U* test. Statistical significance was determined based on *p*‐values: *p* < 0.05 ( ^∗^), *p* < 0.01 ( ^∗∗^), and *p* < 0.001 ( ^∗∗∗^); ns indicates no statistically significant difference (*p* ≥ 0.05).

To further evaluate the breadth of Tfh and Tfr responses, we compared peak responses across three vaccines: inactivated influenza, mRNA COVID‐19, and live‐attenuated measles vaccines. Both influenza and measles vaccinees displayed comparable frequencies of Tfh and Tfr cells (Figure 7B), supporting previous findings that inactivated and live‐attenuated vaccines can generate balanced follicular responses [8]. In contrast, recipients of COVID‐19 mRNA vaccines exhibited high Tfh cell frequencies but significantly lower Tfr frequencies (Figure 7B). This imbalance may suggest enhanced helper activity with reduced regulatory oversight, potentially influencing the quality and duration of humoral immunity [35, 36].

These findings highlight that vaccine platform and formulation play a critical role not only in shaping the magnitude of Tfh responses but also in modulating the regulatory mechanisms that govern the follicular immune balance.

## 4. Discussion

This study shows that an extended AIM assay based on OX40, CD25, CXCR5, Bcl6, and FoxP3 coexpression effectively identifies antigen‐specific circulating memory Tfh and Tfr cells following in vitro recall with influenza, SARS‐CoV‐2, or measles antigens. The detected responses represent bona fide memory OX40^+^ populations, as cells were reactivated ex vivo days after influenza vaccination, months after COVID‐19 mRNA vaccination, and even years after measles immunization. Influenza, COVID‐19, and measles vaccines all induced sustained humoral immunity, with the influenza cohort additionally showing increased frequencies of CD38^+^CD27^+^CD39^+^ memory B cells. Functional assays in the influenza cohort confirmed that sorted antigen‐specific Tfh cells (OX40^+^CD25^+^CXCR5^+^PD‐1^+^Bcl6^+^) promoted memory B‐cell expansion and specific antibody production, accompanied by elevated CXCL13, IL‐21, APRIL, and sCD40L secretion.

The inactivated influenza vaccine provided a robust model to study recall responses in previously exposed individuals. As expected, we observed sustained antibody titers by day 28 postvaccination, correlating with the expansion of memory B cells and consistent with established correlates of protection [32, 37]. This was paralleled by transient peaks of activated CD4^+^ T cells and Tfh (CD45RO^+^CXCR5^+^CCR7^low^PD‐1^+^) cells around day 7 (not shown), in agreement with prior studies [26, 38]. While circulating Tfh populations can include bystander‐activated cells responding to inflammatory cytokines [39, 40], the AIM assay overcomes this ambiguity by specifically identifying antigen‐reactive subsets within the broader Tfh pool.

The OX40/CD25 AIM strategy, validated for detecting antigen‐specific CD4^+^ T cells [29–31], allowed precise discrimination of functionally distinct follicular subsets, including Tfh (OX40^+^CD25^+^CXCR5^+^FoxP3^−^), Tfr (OX40^+^CD25^+^CXCR5^+^FoxP3^+^), and conventional Tregs (OX40^+^CD25^+^CXCR5^-^FoxP3^+^). The shared FoxP3 expression between Tfr and Tregs supports a common lineage origin and aligns with their regulatory role in GC responses [41–43].

Although IL‐2 signaling is classically known to antagonize Tfh cell differentiation through repression of Bcl6, thereby limiting GC formation [44, 45], several studies have shown that antigen‐specific Tfh cells can nonetheless transiently express CD25 following activation. This apparent paradox reflects the dynamic regulation of IL‐2 responsiveness across Tfh subsets and differentiation stages. GC Tfh cells actively restrict IL‐2 signaling to preserve their phenotype and function (18), whereas circulating and memory Tfh populations retain greater plasticity and can transiently upregulate CD25 upon TCR engagement, likely supporting short‐term survival, activation, or homeostatic proliferation [32, 46]. The development of AIM assays further demonstrated that antigen‐specific human Tfh cells can be sensitively identified by upregulation of CD25 and OX40 following stimulation, highlighting CD25 as a marker of recent activation rather than sustained IL‐2 responsiveness [31]. Moreover, IL‐2 exerts context‐dependent effects, where high levels suppress Tfh differentiation, while lower levels may permit their maintenance and coexistence with Tfr cells that tightly control GC responses [19, 47]. Collectively, these findings support the view that CD25 expression on antigen‐specific Tfh cells reflects a transient activation state within a finely tuned balance between IL‐2‐mediated signaling, lineage stability, and functional adaptability in the follicular memory compartment.

Antigen‐specific Tfh and Tfr cells expressed Bcl6 following recall stimulation, confirming their follicular identity. Contrary to earlier reports that circulating Tfh cells lack Bcl6 [48], our results indicate that memory Tfh cells retain this transcriptional signature, supporting their functional competence. Their ability to secrete IL‐21, CXCL13, sCD40L, and APRIL [49] further underscores their capacity to promote B‐cell differentiation and GC reactivation [50]. The coexpression of T‐bet suggests a Th1‐skewed phenotype, typical of antiviral responses [26, 38, 47]. Interestingly, both Tfh and Tfr subsets produced IL‐21, suggesting that in humans, this cytokine supports rather than antagonizes Tfr differentiation, in contrast to murine models (51,52). Increased CXCL13 secretion in cocultures indicates that circulating antigen‐specific Tfh cells retain the capacity to migrate to lymphoid follicles and rekindle GC activity, likely contributing to transient plasmablast bursts observed postvaccination [32].

Comparative analyses across vaccine platforms revealed differences in the coordination of helper and regulatory follicular responses. In influenza vaccinees, Tfh and Tfr frequencies rose synchronously, peaking at day 7 and contracting by day 28, consistent with tightly regulated GC dynamics [32, 41]. In contrast, mRNA COVID‐19 vaccination was associated with a strong expansion of Tfh cells with comparatively limited Tfr induction, resulting in an elevated Tfh:Tfr ratio [34, 35]. Cross‐sectional analyses at peak response further supported a balanced Tfh‐Tfr profile following influenza and measles vaccination, whereas mRNA vaccination favored Tfh predominance.

Together, these observations support the concept that vaccine platforms may differentially engage immune pathways that influence the balance between Tfh and Tfr cells and thereby modulate the GC output. Such platform‐dependent cues may shape both the magnitude and qualitative features of GC reactions. Indeed, GC dynamics rely on the interplay of stimulatory signals from Tfh cells and suppressive inputs from Tfr cells to optimize affinity maturation while preserving clonal diversity [36, 51].

Although most human vaccine studies have focused primarily on Tfh responses, the regulatory follicular compartment remains comparatively underexplored despite its essential role in shaping GC dynamics and maintaining antibody quality [18, 41]. In this context, the extended AIM assay represents a practical and minimally invasive approach to simultaneously monitor Tfh and Tfr populations in humans, offering a scalable framework to investigate the follicular immune balance across vaccination settings.

Integrating such analyses into future studies, including those with matched antigens and longitudinal intraindividual designs, will be valuable to further delineate the relative contributions of the vaccine platform, antigenic context, and host factors. Collectively, these findings highlight the importance of considering both helper and regulatory follicular compartments to achieve a more comprehensive understanding of vaccine‐induced immunity and to inform the development of strategies that elicit potent yet appropriately regulated long‐term antibody responses.

## 5. Limitations

Several limitations should nevertheless be considered when interpreting these findings. The comparisons across vaccine platforms were performed using distinct cohorts that differed in age distribution, prior antigen exposure, and pathogen‐specific immune history, all of which are known to shape baseline immunity and recall responses. Although these factors are unlikely to fully account for the distinct patterns observed, they may have contributed to the magnitude and kinetics of the measured responses. Notably, age alone does not readily explain the reduced Tfr representation, as studies in aged models report preserved or increased regulatory follicular compartments [52–54], and the robust Tfh responses and antibody production observed in the COVID‐19 cohort indicate maintained functional competence. Future studies using matched antigens and longitudinal intraindividual designs will therefore be important to more precisely define the relative contributions of the vaccine platform, antigenic context, and host‐related factors to follicular immune regulation.

## Author Contributions

The study was conceived by N. Seddiki. Participants were recruited and coordinated by C. Tcherakian and F. Tangy with the help of V. Godot and N. Seddiki. Sample handling and processing were performed by A. China, A. Rahman, L. Hani, M. Kumamoto, Mathieu Surenaud, and T. Nogimori. The methodology was developed by N. Seddiki, A. China, A. Rahman, L. Hani, M. Kumamoto, Mathieu Surenaud, and T. Nogimori. The work was supervised by N. Seddiki and T. Yamamoto. Funding was acquired by N. Seddiki and T. Yamamoto. The original draft was written by N. Seddiki. The manuscript was edited by M. Kumamoto, Mathieu Surenaud, V. Godot, T. Nogimori, J. D. Lelièvre, Y. Levy, F. Tangy, T. Yamamoto, and N. Seddiki.

## Funding

This study was funded by the Chaire d’Excellence from INSERM/University of Paris‐Est‐Créteil and the Japanese Society for the Promotion of Science Grant‐in‐Aid for Scientific Research (B) (Grant 24K02498).

## Conflicts of Interest

The authors declare no conflicts of interest.

## Supporting Information

Additional supporting information can be found online in the Supporting Information section.

## Supporting information


**Supporting Information** S1. Memory B cells (CD3^−^CD19^+^, CD38^+^CD27^+^, and CD38^+^CD39^+^) were measured by flow cytometry at baseline and day 3, 7, 14, and 28 post flu‐vaccination. S2. Gating strategy and frequencies of influenza‐specific CD25^⁺^CD134^⁺^ CD4^⁺^ T cells, including Tfh and Tfr subsets for five vaccinated individuals.

## Data Availability

The data that support the findings of this study are available from the corresponding author upon reasonable request.
