# Supplementary material for: Dynamics of Circulating Follicular Helper and Regulatory T‐Cell Memory Induced by mRNA, Inactivated, and Live‐Attenuated Vaccines
Source: J Immunol Res. 2026 Jul 8;2026:2293380. doi: 10.1155/jimr/2293380 (PMC13342843; doi:10.1155/jimr/2293380)
Supplement: Supplementary file 1 — Supporting Information S1. Memory B cells (CD3−CD19+, CD38+CD27+, and CD38+CD39+) were measured by flow cytometry at baseline and day 3, 7, 14, and 28 post flu‐vaccination. S2. Gating strategy and frequencies of influenza‐specific CD25⁺CD134⁺ CD4⁺ T cells, including Tfh and Tfr subsets for five vaccinated individuals. [file JIMR-2026-2293380-s001.pptx]

## Slide 1
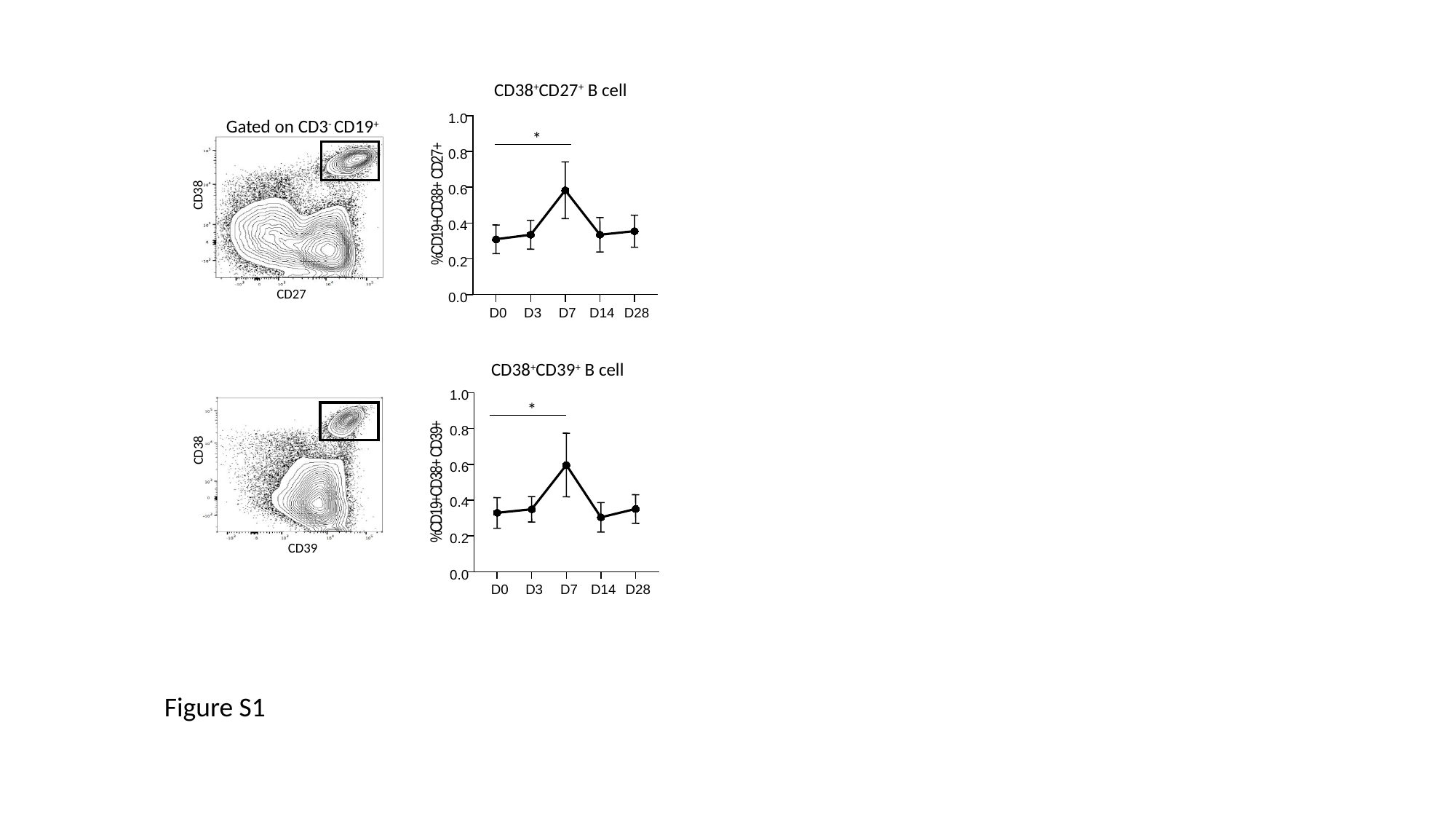

CD38+CD27+ B cell
Gated on CD3- CD19+
CD38
CD27
1.0
+
7
0.8
2
D
C
+
0.6
8
3
D
C
+
0.4
9
1
D
C
%
0.2
0.0
D0
D3
D7
D14
D28
*
CD38+CD39+ B cell
1.0
+
9
0.8
3
D
C
+
0.6
8
3
D
C
+
0.4
9
1
D
C
%
0.2
0.0
D0
D3
D7
D14
D28
*
CD38
CD39
Figure S1

## Slide 2
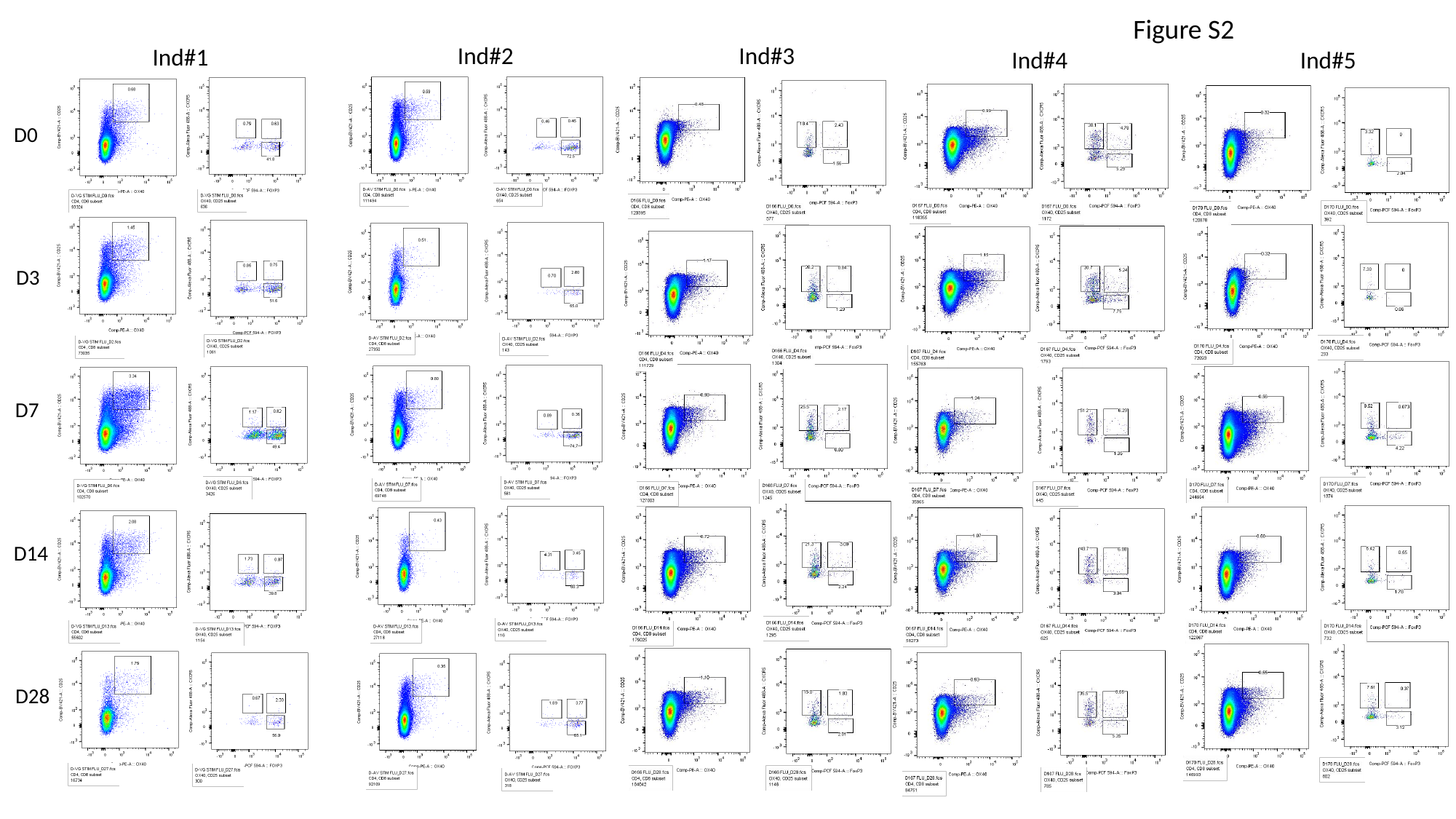

Figure S2
Ind#2
Ind#3
Ind#1
Ind#4
Ind#5
D0
D3
D7
D14
D28
